# Pericoronitis among dental students: experience, awareness, and management practices

**DOI:** 10.3389/fdmed.2026.1774408

**Published:** 2026-03-17

**Authors:** Ghita Elbasraoui, Zineb Loubaris, Youssra Azzouz, Saliha Chbicheb

**Affiliations:** 1 Department of Oral Surgery, International University of Rabat, College of Health Sciences, Health Science Research Center (CReSS), International Faculty of Dental Medicine, Technopolis Parc, Sala-Al Jadida, Morocco; 2 Department of Oral Surgery, Faculty of Dentistry, Mohammed V University, Rabat, Morocco

**Keywords:** cross-sectional studies, Morocco, pericoronitis, students, dental, surveys and questionnaires

## Abstract

**Introduction:**

Pericoronitis is a common inflammatory condition associated with partially erupted third molars and frequently encountered in dental practice. Understanding dental students’ experience and management approaches is important for improving education and clinical decision-making.

**Objective:**

To assess the prevalence, clinical experience, and management practices related to pericoronitis among undergraduate dental students.

**Methods:**

A cross-sectional questionnaire-based study was conducted among clinical dental students. Data were analyzed descriptively using frequencies and percentages. Associations between categorical variables were assessed using the Chi-square test with significance set at *p* < 0.05.

**Results:**

A total of 259 students participated. Among them, 35.5% were sixth-year students, 32% in the fifth year, and 31.5% in the fourth year. History of pericoronitis was reported by a substantial proportion of students. A statistically significant association was found between history of pericoronitis and dental consultation (*χ*^2^ = 16.67, *p* < 0.001). No significant association was observed between sex or academic year and history of pericoronitis.

**Conclusion:**

Personal experience with pericoronitis significantly influences dental consultation behavior among students. These findings highlight the importance of strengthening preventive education and evidence-based management strategies in undergraduate dental training.

## 1 Introduction

Pericoronitis is a common inflammatory condition affecting the soft tissues surrounding the crown of a partially erupted tooth, most frequently the mandibular third molar. It typically arises when a gingival operculum partially covers the erupting tooth, creating a favorable niche for plaque accumulation and bacterial growth, leading to local infection and inflammation (1, 2). Clinically, pericoronitis may present in a congestive form, characterized by erythema, edema, and mild discomfort, or evolve into a suppurative form associated with severe pain, trismus, halitosis, fever, lymphadenopathy, and potential spread to adjacent anatomical spaces (3). The condition predominantly affects adolescents and young adults, particularly between 17 and 25 years of age, corresponding to the eruption period of third molars (4). Several local and systemic factors contribute to its development, including inadequate oral hygiene, tooth angulation and impaction depth, occlusal trauma from the opposing tooth, stress, smoking habits, and host immune response (5). Recurrent episodes are common and may significantly impair quality of life and academic or professional performance. Pericoronitis is frequently considered a dental emergency due to the intensity of pain and the risk of infectious complications. Management strategies vary widely and may include local irrigation, antiseptic mouth rinses, analgesics, anti-inflammatory drugs, antibiotic therapy, and surgical approaches such as operculectomy or extraction of the offending tooth (6). However, inappropriate self-medication, delayed dental consultation, and unjustified antibiotic prescription are commonly reported, particularly among young adults, raising concerns regarding recurrence, treatment failure, and antimicrobial resistance (7). Dental students represent a unique population for investigating pericoronitis, as they are expected to possess higher levels of oral health knowledge while simultaneously being exposed to academic stress and irregular health-seeking behaviors. Despite this, studies have shown that even healthcare students may underestimate the severity of pericoronitis or rely on symptomatic treatment rather than seeking definitive care (8). Evaluating their experiences and management practices is essential, as these students are future practitioners whose attitudes will influence clinical decision-making and patient education. Although pericoronitis has been extensively described in clinical and surgical literature, epidemiological data focusing on its prevalence, clinical presentation, recurrence patterns, and management among dental students remain limited, particularly in North African and developing country contexts. Identifying gaps in knowledge and practice within this population may help improve undergraduate training and promote evidence-based management strategies.

Therefore, the aim of the present study was to assess the prevalence, clinical manifestations, recurrence characteristics, and management approaches of pericoronitis among 4th-, 5th-, and 6th-year dental students at the International Faculty of Dental Medicine of Rabat. By analyzing socio-demographic characteristics, lifestyle habits, and therapeutic behaviors, this study seeks to contribute to a better understanding of pericoronitis management among future dental professionals and to support the development of targeted educational interventions.

## 2 Methods

### 2.1 Study design

This cross-sectional descriptive study was conducted between September and November 2025. A digital survey assessing socio-demographic characteristics, lifestyle habits, and history of pericoronitis was administered to undergraduate dental students enrolled in the 4th, 5th, and 6th academic years at the International Faculty of Dental Medicine of Rabat (Morocco). All eligible students were invited to participate. Participation in the study was voluntary, and anonymity and confidentiality. The questionnaire was self-administered and distributed electronically to facilitate participation and minimize response bias.

The study was conducted in accordance with the principles of the Declaration of Helsinki and was approved by the Institutional Review Board of the International Faculty of Dental Medicine of Rabat. Informed consent was obtained electronically from all participants prior to their inclusion in the study.

### 2.2 Questionnaire

A digital questionnaire was developed based on relevant literature and reviewed by three senior dental residents to ensure clarity and content relevance. A pilot evaluation was performed before final distribution. The questionnaire was distributed electronically using Google Forms. The questionnaire was structured into three main sections. The first section collected socio-demographic data, including gender, age, academic year of study (4th, 5th, or 6th year), and medical history. The second section focused on lifestyle habits and oral hygiene practices, including smoking status, alcohol consumption, and toothbrushing frequency. The third section assessed the history and clinical characteristics of pericoronitis. Participants were asked about the occurrence of pericoronitis episodes, age at first episode, number of recurrences, and whether the condition was perceived as a dental emergency. Clinical manifestations were evaluated through multiple-choice questions addressing gingival inflammation and swelling, pain, trismus, halitosis, fever, lymphadenopathy, and tooth mobility.

Additional questions explored health-seeking behavior and management strategies during acute episodes, including dental consultation, self-medication practices, and treatments used (analgesics, mouth rinses, antibiotics, and anti-inflammatory drugs). Participants were also asked about the management of affected third molars, including absence of treatment, delayed extraction, or immediate extraction, as well as patterns of antibiotic use (prophylactic, curative, both, or none).

### 2.3 Data management

Collected data were coded and entered a spreadsheet prior to analysis. Socio-demographic variables, lifestyle habits, and clinical characteristics of pericoronitis were treated as categorical variables and summarized using frequencies and percentages. Age was recorded as a continuous variable and categorized into age groups for descriptive purposes. Data related to pericoronitis history included the presence or absence of episodes, age at first occurrence, number of episodes, perceived urgency of the condition, and reported clinical signs and symptoms. These variables were analyzed descriptively and expressed as percentages. Management-related variables, including health-seeking behavior, type of treatment used during acute episodes (analgesics, mouth rinses, antibiotics, and anti-inflammatory drugs), management of the affected third molars (no treatment, delayed extraction, or immediate extraction), and patterns of antibiotic use (none, prophylactic, curative, or both), were coded as categorical variables and presented as proportions. To facilitate statistical analysis, responses allowing multiple selections were analyzed by calculating the proportion of participants reporting each option. Missing or incomplete responses were excluded from the analysis on a variable-by-variable basis.

### 2.4 Statistics

Data was automatically recorded on Google Sheets and processed on Excel and analysed in JAMOVI software version 2.4.12. Quantitative data were presented as mean and standard deviation, and qualitative data as numbers and percentages. Descriptive statistics were calculated as frequencies and percentages. Associations between categorical variables (sex, academic year, history of pericoronitis, consultation behavior, and management practices) were evaluated using the Chi-square test. A *p*-value <0.05 was considered statistically significant.

## 3 Results

### 3.1 Participant characteristics

All eligible students (*n* = 496) were invited to participate. A total of 259 completed the questionnaire, corresponding to a response rate of 52.2%. The study population included 88 males (34.0%) and 171 females (66.0%), corresponding to a sex ratio of 0.51. Most respondents were aged 20–22 years (*n* = 172; 66.4%), followed by those aged 23–25 years (*n* = 84; 32.4%), while only three participants were older than 25 years (1.2%). Regarding academic level, students from the 4th, 5th, and 6th years accounted for 32.4% (*n* = 84), 32.1% (*n* = 83), and 35.5% (*n* = 92) of the sample, respectively.

Most participants reported no underlying systemic conditions (*n* = 222; 85.7%). Concerning lifestyle habits, most students were non-smokers (*n* = 240; 92.7%), whereas 19 (7.3%) reported tobacco use. Similarly, alcohol consumption was uncommon, with 245 students (94.6%) reporting no alcohol intake and 14 (5.4%) indicating alcohol use. Oral hygiene practices varied among respondents: toothbrushing was performed once daily by 16 participants (6.2%), twice daily by 151 (58.3%), and three times per day by 85 (32.8%) (Table 1).

**Table 1:** Characteristics of the population.

Characteristics	Values *N* = 259
Age (Years)^a^	
20–22	172 (66.4%)
23–25	84 (32.4%)
>25	3 (1.2%)
Gender^a^	
Male	88 (34.00%)
Female	171 (66.00%)
Year of study^a^	
4^th^	84 (32.4%)
5^th^	83 (32.1%)
6^th^	92 (35.5%)
Lifestyle habits^a^	
Nonsmoking	240 (29.7%)
Smoking	19 (7.3%)
Nonalcoholic	245 (94.6%)
	14 (5.4%)
Toothbrushing^a^	
Once/day	16 (6.2%)
Twice/day	151 (58.3%)
Three/ day	85 (32.8%)

a Expressed as count (percentage).

### 3.2 Students’ knowledge

**Figure 1:**
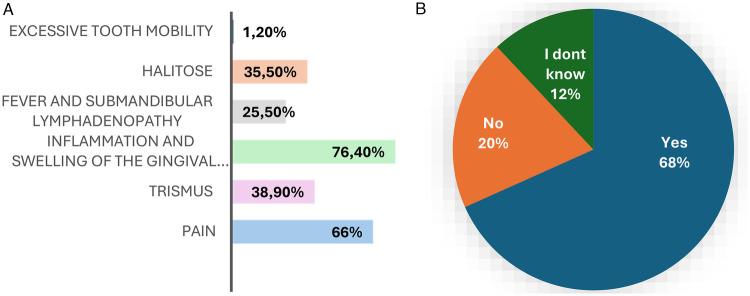
Students’ knowledge. **(A)** Recognition of clinical features of pericoronitis among dental students. **(B)** Students’ perception of pericoronitis as a dental emergency.

Students’ knowledge of pericoronitis was assessed through their recognition of its clinical features and their perception of its urgency. Regarding clinical presentation, pain was identified as a sign of pericoronitis by 172 participants (66.4%), while trismus was reported by 93 students (35.9%). Inflammation and swelling of the gingival operculum surrounding the impacted third molar were the most frequently recognized features, selected by 198 respondents (76.4%). Fever and submandibular lymphadenopathy were identified by 66 students (25.5%), whereas halitosis was considered a clinical sign by 92 participants (35.5%). Excessive tooth mobility was less frequently associated with pericoronitis (3, (1.2%) (*p* < 0.05) (Figure 1A).

Concerning the perceived severity of the condition, 177 participants (68.3%) considered pericoronitis to be a dental emergency. In contrast, 51 students (19.7%) did not regard it as an emergency, while 31 respondents (12.0%) reported being unsure whether pericoronitis constitutes a dental emergency (Figure 1B).

### 3.3 Students’ experience with pericoronitis

A total of 134 students (51.7%) reported having experienced at least one episode of pericoronitis. Among them, 24 participants (15.5%) reported that the first painful episode occurred before the age of 18 years, whereas the majority experienced their first episode between 18 and 21 years of age (*n* = 114; 73.5%). Seventeen students (11.0%) reported the onset of pericoronitis between the ages of 22 and 25 years. Regarding recurrence, most affected participants reported one to two painful episodes (*n* = 95; 61.3%), while 46 students (29.7%) experienced three to five episodes, and 14 (9.0%) reported more than five episodes. No statistically significant association was found between sex and history of pericoronitis (*p* > 0.05). Similarly, no significant association was observed between academic year and pericoronitis occurrence (*χ*^2^ = 4.31, *p* = 0.116).

Concerning clinical manifestations, gingival inflammation was the most frequently reported sign among students with a history of pericoronitis (*n* = 118; 75.2%), followed by pain localized around the causative wisdom tooth (*n* = 107; 68.2%). Difficulty in mouth opening, halitosis, fever, and swelling were reported by 21 (17.2%), 26 (16.6%), 18 (11.5%), and 1 (0.6%) participants, respectively. (Table 2).

**Table 2:** Dental students’ experience with pericoronitis.

Episode of pericoronitis	%
Yes	51,70%
No	48,30%
Age of the first painful episode	%
<18 years	15,50%
18–21 years	73,50%
22–25 years	11%
>25 years	0%
Number of episodes of pericoronitis	%
1 to 2 times	61,30%
3 to 5 times	29,70%
>5 times	9%
Clinical manifestations	%
Gingival inflammation	75,20%
pain around the wisdom teeth	68,20%
Trismus	17,20%
Halitosis	16,60%
Fever	11,50%
Swelling	0,60%

### Management of pericoronitis

**Figure 2:**
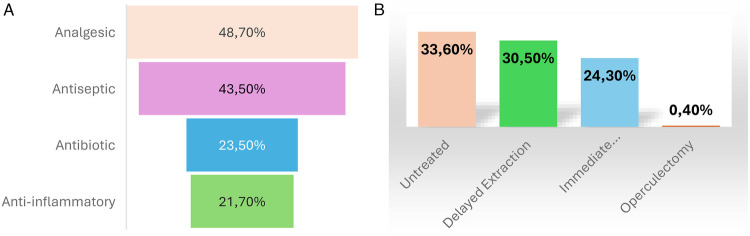
Management of pericoronitis. **(A)** Treatment modalities used during acute pericoronitis episodes. **(B)** Management of the causative third molar in pericoronitis.

During acute episodes of pericoronitis, dental consultation was sought by 71 participants (27.4%). Among those who consulted, analgesics were the most frequently prescribed treatment (*n* = 56; 48.7%), followed by antiseptic mouth rinses (*n* = 50; 43.5%). Antibiotics and anti-inflammatory drugs were prescribed to 27 (23.5%) and 25 (21.7%) participants, respectively. Regarding the management of the causative third molar, the tooth was left untreated in 87 cases (33.6%). Delayed extraction was performed in 79 participants (30.5%), whereas immediate extraction was carried out in 63 cases (24.3%). Operculectomy was reported in only one case of pericoronitis. (Figure 2). A statistically significant association was found between history of pericoronitis and dental consultation (*χ*^2^ = 16.67, *p* < 0.001).

## 4 Discussion

### 4.1 Participant characteristics

The present survey involved 259 undergraduate dental students, yielding a response rate of 52.2%, which is in line with participation levels reported in comparable questionnaire-based investigations among dental undergraduates (9). The marked predominance of women (66.0%) confirms the well-established feminization of dentistry (10). The age profile of the participants was largely concentrated in early adulthood, with nearly all respondents under 25 years of age. This pattern corresponds to the typical demographic composition of students in the clinical phases of dental training (11). Such age homogeneity is particularly relevant, as pericoronitis predominantly affects individuals during late adolescence and young adulthood, coinciding with the eruption of third molars (12). Lifestyle behaviors reported in this study revealed limited engagement in tobacco and alcohol use, a trend that has also been observed in dental student populations from regions with strong preventive health orientations (13). Professional awareness of the harmful effects of these habits on oral and systemic health may partly explain this observation. Regarding oral hygiene, most participants reported brushing their teeth at least twice daily, in accordance with international recommendations and findings from previous studies indicating superior oral health behaviors among dental students compared with the general population (14). Taken together, the demographic characteristics, health status, and behavioral patterns observed in this study are consistent with those reported in the literature and provide a solid framework for interpreting students’ knowledge, experiences, and management of pericoronitis.

### 4.2 Students’ knowledge

Pain and inflammation of the gingival operculum were the most frequently identified signs, in accordance with the literature describing them as the hallmark manifestations of pericoronitis (15, 16). This level of awareness is expected among 4th-, 5th- and 6th-year students, who are regularly exposed to third molar pathology during clinical training. In contrast, systemic and functional signs such as trismus, fever, and submandibular lymphadenopathy were less frequently recognized, suggesting an underestimation of disease severity. These manifestations are well documented in advanced or suppurative forms and may indicate progression toward deeper or systemic infections if left untreated (17, 18). Halitosis was moderately acknowledged, consistent with its association with anaerobic bacterial activity beneath the operculum (19). Regarding clinical severity, more than two-thirds of participants considered pericoronitis to be a dental emergency, reflecting appropriate clinical judgment given the potential for rapid progression and complications (20).

### 4.3 Students’ experience with pericoronitis

The experience of pericoronitis reported by more than half of the surveyed dental students highlights the high frequency of this condition among young adults, particularly those in the clinical years of dental education. This prevalence is consistent with epidemiological data indicating that pericoronitis most commonly affects individuals during late adolescence and early adulthood, coinciding with the eruption period of mandibular third molars (21, 22). The predominance of first painful episodes occurring between 18 and 21 years further supports this well-established temporal association (23). Regarding recurrence, most affected students experienced one to two episodes; however, a substantial proportion reported multiple recurrences. Recurrent pericoronitis is a well-documented phenomenon and has been associated with persistent local etiological factors such as partial tooth eruption, plaque retention beneath the operculum, occlusal trauma, and inadequate local management (24, 25). The experiential burden of pericoronitis among dental students should not be underestimated, as repeated painful episodes may affect quality of life, academic performance, and clinical attendance. Previous studies have shown that personal experience with oral diseases can influence students’ clinical attitudes, decision-making processes, and future patient management strategies (26). No association was observed between sex or academic year and the occurrence of pericoronitis, suggesting that the condition may depend more on anatomical factors, oral hygiene, or eruption patterns than demographic characteristics or level of training (22).

### 4.4 Management of pericoronitis

Less than one-third of participants sought dental consultation during acute episodes, suggesting a tendency toward self-management or underestimation of the condition. Similar findings have been reported in studies among young adults and dental students (27, 28). Among students who consulted a dentist, analgesics and antiseptic mouth rinses were the most frequently prescribed treatments. This approach is consistent with current recommendations for the initial management of mild or congestive forms of pericoronitis (29). Antibiotics were prescribed to nearly one-quarter of participants, while anti-inflammatory drugs were also commonly used. The prescription patterns observed in this study highlight persistent challenges in antibiotic stewardship (30). Regarding definitive management, a substantial proportion of causative third molars were either left untreated or managed by delayed extraction. While postponing extraction may be justified in certain acute inflammatory phases, the persistence of the etiological factor exposes patients to recurrent episodes (31). Immediate extraction, performed in approximately one-quarter of cases, has been shown to be safe and effective when appropriate clinical conditions are met, potentially reducing recurrence and improving patient outcomes (32). The very low rate of operculectomy observed in this study is consistent with the declining popularity of this procedure, given its association with high recurrence rates compared with extraction (33).

## 5 Conclusion

Pericoronitis remains a frequent and potentially recurrent condition related to third molar eruption, with possible functional and infectious complications if not adequately managed. Despite the availability of well-established clinical guidelines, its management continues to vary in practice. The present study highlights existing gaps between knowledge and behavior among undergraduate dental students, particularly regarding timely consultation, recurrence prevention, and definitive treatment strategies. The persistence of recurrent episodes and the predominance of conservative approaches underscore the need for improved translation of theoretical knowledge into clinical decision-making. From an educational perspective, these findings support the integration of structured, evidence-based training on pericoronitis management within undergraduate curricula, with emphasis on early intervention and rational therapeutic choices. Future research should focus on evaluating the impact of targeted educational strategies on students’ clinical practices and long-term patient outcomes.

## Data Availability

The original contributions presented in the study are included in the article/Supplementary Material, further inquiries can be directed to the corresponding author.
